# Omnichannel Strategy and Consumer Behavior in Distribution Channels: Trends in the Ophthalmology Sector

**DOI:** 10.3389/fpsyg.2020.01142

**Published:** 2020-06-03

**Authors:** Lourdes Rivero Gutiérrez, Rocio Samino García

**Affiliations:** Department of Business Administration, Faculty of Social Sciences and Law, Rey Juan Carlos University, Madrid, Spain

**Keywords:** ophthalmology sector, supply chain, distribution channels, omnichannel, consumer behavior

## Abstract

Changes in consumer behavior are forcing companies to rapidly shift their distribution channels toward an omnichannel model. In the case of the ophthalmology sector, however, the purchase of contact lenses and prescription glasses requires professional examination by a trained eye specialist. This peculiarity of the sector affects the shift toward omnichannel. This paper is novel in that it addresses a research gap by studying the distribution channels for regulated products and services such as health products that required a closer professional service. This paper addresses the transformation of companies toward an omnichannel model using a common scientific forecasting method (i.e., the Delphi method) to identify trends and problems. In this scenario of high complexity and uncertainty where there is insufficient relevant quantitative information for decision making, the Delphi method is applied to ensure a consensual decision-making process. Consensus was reached by a panel of 11 experts on the Spanish ophthalmology sector after three rounds of questioning. The final set of indicators involve 19 different criteria grouped into four categories (1) current situation of the distribution channel, (2) changes in distribution channels, (3) trends and near future of the distribution channel, and (4) consequences and adaptations for supply chain agents. The results from each round of consultation were then analyzed by means of statistical analysis with SPSS and discussed for each category. The results confirm that traditional intermediaries face difficulties to avoid being squeezed out of the sector because of shortening distribution channels and the entry of new online intermediaries with service integration models. We identify future scenarios and define actions that different agents can take to enhance their competitiveness in the short and long term. The arrival of omnichannel distribution is inevitable in the ophthalmology sector, entailing a major transformation from a rigid traditional distribution model to a more complex and flexible model following the entry of new online intermediaries.

## Introduction

In recent years, consumer behavior and habits have changed drastically, leading to the transformation of most, if not all, markets. The main driver of these changes has been the Internet. Online sales and e-commerce, among other factors, have changed how people consume services and goods around the world.

Customers used to visit brick-and-mortar stores to gather information with the help of salespeople to find what they wanted or needed, often concluding their shopping there (Mármol and Fernández, 2019). Physical stores were one of the few available sources of product information but nowadays the arrival of digital devices has increased the number of sources of information. Consumers nowadays tend to combine these digital services with brick-and-mortar establishments to purchase products and services and to contact firms for other purposes, including requesting information, soliciting technical advice, providing feedback about products and services and inquiring about a product’s use or availability. As the new channels such as internet and mobile rise and are offered to customers, the customer experience becomes even more digitalized (Nash et al., 2013; Kang et al., 2019) and results in an increase in the number and complexity of customer–firm interactions (Melero et al., 2016). Moreover, customer goes in and out of different channels to use the benefits of each channel offers rather than staying with one type of channel, what is known as omnichannel behavior (Verhoef et al., 2007, 2015; Kang et al., 2019). Omnis is a Latin word meaning “all” or “universal,” so omnichannel means “all channels together” (Mármol and Fernández, 2019). Omnichannel shopping offers an integrated strategy where the different channels interact with each other and are used simultaneously in both the process of information seeking and the process of purchasing (Puccinelli et al., 2009). This perfectly integrated system of online channels and physical stores can improve the performance of these channels as well as the customer’s purchase experience (Puccinelli et al., 2009; Rigby, 2011; Kang et al., 2019).

However, Ailawadi and Farris (2017) notes that the use of an integrated system is still hard for both upstream suppliers and downstream retailers. Digital transformation affects not only online transactions but also transactions made through offline channels. On one hand, Kang et al. (2019) explain that the offline channel is facing with dramatic decline while online channel through various digital channel such as mobile sales shows significant growth. This effect can be due to online distribution has grown through the cannibalization of brick-and-mortar stores (Mármol and Fernández, 2019). From the perspective of traditional channel point of view, such situation is considered crisis (Sopadjieva et al., 2017). In this context of crisis, there are some studies progressing about ways to strengthening the competitiveness of offline retail through specializing its own uniqueness (Sopadjieva et al., 2017; Fisher et al., 2018).

On the other hand, some experts argue that both offline and online channels are able to be consistent, concurrent and compatible (Gabisch and Gwebu, 2011; Melero et al., 2016) and coexist due to physical establishments are mutating under digital transformation, changing the way customers shop nowadays (Gallino and Moreno, 2014; Mármol and Fernández, 2019). Melis et al. (2015) and Melero et al. (2016) highlight the importance to understand what drive customers to every channel so that companies can offer them a satisfactory purchase experience.

The co-existence of physical stores, e-commerce and m-commerce shows that omnichannel strategies are well established in some markets such as clothing. However, certain sectors have structures that are more complex, so they find it less easy to adapt. Examples are dental clinics, pharmacies and vision centers. In the ophthalmology sector, vision centers remain the first choice today because new consumers in the digital era still value certain attributes that belong exclusively to physical stores: touching and trying products and interacting with staff. The ophthalmology sector is a mature and competitive market that consists of all activities related to eye care. Some treatments require eyeglasses or contact lenses (primarily farsightedness, myopia, and presbyopia), while other treatments do not require eyeglasses (e.g., cataracts or amblyopia). The most profitable treatments for vision centers and eye care professionals are treatments of conditions that require eyeglasses or contact lenses because the sale of glasses and contact lenses represents a large proportion of vision centers’ income. There are many reasons why the need for eye care will grow in the future, such as an aging population (Morgan and Efron, 2009) and the greater use of electronic devices with small screens such as tablets and smartphones (Ward, 1987; Moulakaki et al., 2018; Bernal-Molina et al., 2019).

According to General Council of College of Optics-Optometrics (GCCOO), (2019), 47% of European youths (25–29 years old) suffered from myopia in 2015, and this percentage is rising. This trend is not only true of Europe. In Spain, with 10,198 opticians and sales revenue of 1.829,91 million euros in 2018 (11% more due to the increase in sales of contact lenses and glasses) is the only European country that has increased the number of optics to cover this demand and improve customer service. For this reason, this paper focuses on the Spanish ophthalmology sector as a relevant case study. Ophthalmology is a health-related sector and a consumer-sensitive business that involves health care professionals and the sale of eyeglasses and contact lenses. This choice is justified by the fact that Spain still has scant legislation governing health care e-commerce, so most companies of ophthalmology sector operating online are acting in a legal vacuum. This sector must be regulated, but in the meantime, businesses in this sector must evolve so as not to be left behind.

The eyewear industry used to depend exclusively on brick-and-mortar stores. However, there has recently been a significant change in consumer behavior. Consumers are becoming increasingly comfortable with online shopping for eyewear. The increasing trend of shopping online for any and every product could be one of the reasons for this change in preferences (Khanna and Bhatia, 2018). According to Brynjolfsson et al. (2013), retailers used to rely on barriers such as geography and customer ignorance to advance their positions in traditional markets. However, technology removes those barriers.

This paper focuses specifically on how the Internet, e-commerce, and users’ changing purchasing behavior affect eyeglass and contact lens distribution channels. In Spain, vision centers cannot operate purely online because they are required to have an eye care professional (optician, ophthalmologist, or optometrist). This requirement presents an opportunity for vision centers. In this exploratory research, the traditional structure of the Spanish ophthalmology sector and the main supply chains that existed in the past are reviewed to analyze how changes in consumer behavior, primarily due to the Internet, have transformed these supply chains. The Delphi method is applied to predict where the sector is evolving based on what has happened in other economies. The results show the consequences and available options that different supply chain agents can choose to adapt, survive, and succeed in this new context of omnichannel distribution and e-commerce.

The paper is structured as follows. Section “Methodology. Delphi Expert Consultation” describes the procedure followed to apply the Delphi method to collect and process the data. Section “Results and Analysis” presents the results and analysis of the Delphi consultation with respect to its 19 criteria grouped into four categories (1) current situation of the distribution channel, (2) changes in distribution channels, (3) trends and near future of the distribution channel, and (4) consequences and adaptations for supply chain agents. Section “Termination Criteria of the Delphi Process” describes the termination criteria of the Delphi process followed by a section “Discussion” of the consensus-based framework. Section “Conclusion” then presents the main conclusions and limitations of the study.

## Methodology: Delphi Expert Consultation

The Delphi method is a technique to structure a group communication process so that the interactions between group members effectively allow a group of individuals to deal with a complex problem (Linstone and Turoff, 1975). The technique is based on the knowledge, opinions, and experiences of the experts and thus does not aim to be representative of a population (Okoli and Pawlowski, 2004). The Delphi method is widely used for forecasting, gathering information for decision-making processes, or, as in this study, obtaining views on possible strategies. Created by the RAND Corporation in the 1950s, the Delphi method has become popular for forecasting in economics (Gordon and Easson, 2005; Kauko and Palmroos, 2014), technology (Gallego and Bueno, 2014), transportation (Allen et al., 2019), social sciences (Jillson et al., 2019), sports (Merkel et al., 2016), and many other disciplines (Gupta and Clarke, 1996; Landeta, 2006; Cerè et al., 2019). It has high predictive accuracy (Rowe and Wright, 1999; Czinkota and Ronkainen, 2005).

According to Linstone and Turoff (1975) and Cabero and Infante (2014), the Delphi method is applicable to this study because (1) no relevant quantitative data are available, (2) available information on the topic is insufficient to solve the problem, (3) the geographical location of the experts does not allow for face-to-face communication, and (4) anonymity of participants is an advantage.

### Panel of Experts

The quality of the results of the Delphi method depends on the combined expertise of all participants. Therefore, improper panel selection is the most serious validity threat in Delphi studies (Mitchell and McGoldrick, 1994; Creswell, 2003). By definition, Delphi panels are not statistically representative but instead contain only the most knowledgeable experts (Powell, 2003). There is no general rule regarding optimal sample size. It depends on the research scope, desired panel heterogeneity, and availability of experts in the field (Fink et al., 1984; Loo, 2002). While many Delphi studies feature 15 to 35 respondents (Gordon, 1992), for surveys with parameters similar to ours (broad scope, homogeneous panel structure, and few available experts), having 5 to 15 participants is defined as optimal (Saaty, 2005).

In this study, an initial group of 18 experts was formed, 11 of whom continued for the duration of the process. The selection of the members of the panel of experts was extremely important. The quality of the selected members was prioritized over the quantity (Powell, 2003). All members were professionals in the ophthalmology industry with at least 3 years’ experience. The combination of experts from different fields provides the advantage to consider problems from different viewpoints (Cerè et al., 2019). To this regard, Table 1 provides a breakdown of the experts’ domain of expertise based on the respondents involved in the tree rounds of consultation.

**TABLE 1:** Distribution of experts by domain of expertise.

Domain of expertise	Initial panel	After 1st round	After 2nd round	After 3rd round	(%)
Manufacturers	5	3	3	3	27
Wholesalers	4	2	2	2	18
Retailers	9	6	6	6	55
Overall	18	11	11	11	100

### Delphi Rounds

**FIGURE 1:**
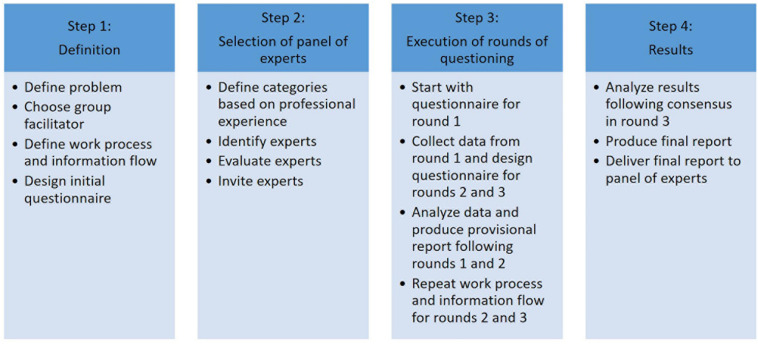
Steps for conducting analysis using the Delphi method.

Three rounds were required to achieve a convergence of opinion on the target of this study. Figure 1 illustrates the steps that must be taken to perform analysis using the Delphi method.

**FIGURE 2:**
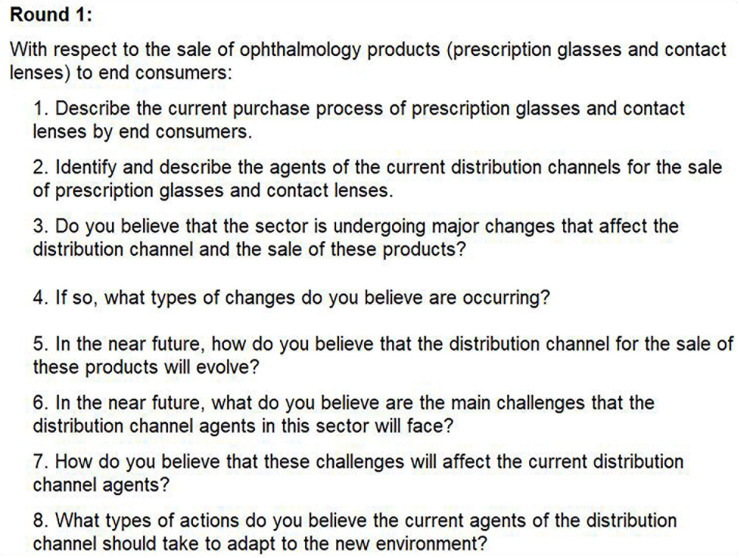
List of questions on the questionnaire used in round 1.

**FIGURE 3:**
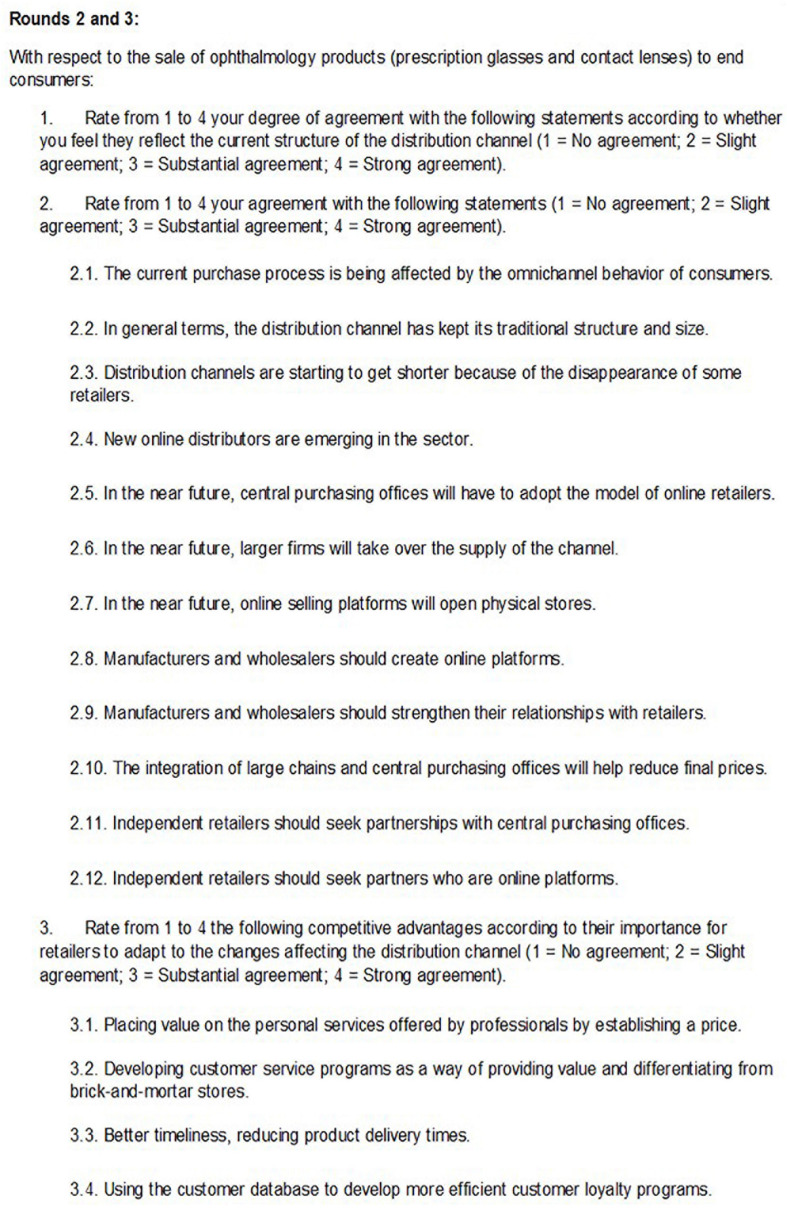
List of questions on the questionnaire used in rounds 2 and 3.

The consultation instrument was a questionnaire. The questions included in the questionnaires issued to the experts in each different round may be consulted in Figures 2, 3.

In the first round, eight open questions were used to collect the diverse opinions of experts. Based on these initial data and the additional observations of the experts, content analysis was performed using NVivo software to identify the most important criteria added by each expert, their similarities, and their frequencies. Based on the 19 criteria identified by this first questionnaire, the second-round questionnaire was developed. The questions in the second-round questionnaire were closed, and experts were asked to accept or not accept each criteria and to score its importance on a four-point Likert scale (from 1 to 4) with no safe neutral option so experts are forced to form an opinion. The experts were also allowed to make observations about their responses.

The results of the second round were shared with the experts in the third round for re-evaluation or ratification of their opinions. The third-round questionnaire comprised four blocks of closed questions based on scales, which the experts used to express their level of agreement or disagreement with respect to the statistical measures that were displayed: (a) mean values for the whole responses; (b) standard deviation for the total dataset; (c) individual response for the former round; and (d) interquartile range (IQR) for the different criteria. After the final round, 11 valid questionnaires were received, and a consensus was reached.

### Consensus

In order to move from one round to the following, it is necessary to establish when an acceptable level of consensus amongst the feedbacks has been achieved. Several consensus measurement strategies are available in the literature (Von der Gracht, 2012). However, the most reliable way of defining consensus is the IQR index (Murphy et al., 1998). According to Rayens and Hahn (2000) and based on a rating scale composed of four points, an IQR ≤ 1 means that the consensus achieved is in a suitable threshold, with 0 corresponding to the strongest value, while the closer it gets to 1, the lower the consensus will be. The standard deviation is used instead as an indicator of the dispersion of the dataset, hence the higher it is, the more scattered are the experts’ responses (Greatorex and Dexter, 2000; Cerè et al., 2019). According to Goldman et al. (2008), standard deviation values greater than 1.5 correspond to a lower consensus. Based on the work by Greatorex and Dexter (2000), mean values are considered as a valid pointer for the importance of the different indicators. There is a debate as to when to stop a Delphi methodology, and based on which assumption. This is discussed in section “Termination Criteria of the Delphi Process.” However, the literature does not provide absolute recommendations, while referring to the “hierarchical stopping criteria” (Von der Gracht, 2012; Cerè et al., 2019) devised by Dajani et al. (1979). The later states that the achievement of the consensus itself (i.e., IQR indicator) is not sufficient to be considered as a stopping criterion, as significant fluctuations might occur between the rounds, and therefore stability is a more reliable concept. This can be assessed as described by English and Kernan (1976) and Cerè et al. (2019) by means of the adoption of the variation coefficient which entails the calculation of the ratio between the standard deviation and mean across all the criteria. This indicator provides a tangible measure of the stability of the system as it advocates that if the difference between the variation coefficients between the two rounds is not significant, it is possible to terminate the process Cerè et al. (2019). The second and third round outputs were processed with Statistical Package for Social Sciences (SPSS).

In this study, the Delphi technique reveals the points of agreement, their degree of consensus, and the hierarchy in terms of their transcendence (Ruiz, 2003). The results of the Delphi method are presented in section “Results and Analysis.”

## Results and Analysis

The final set of indicators involve 19 different criteria grouped into four categories: (1) current situation of the distribution channel, (2) changes in distribution channels, (3) trends and near future of the distribution channel, and (4) consequences and adaptations for supply chain agents.

The feedback from second round provided to experts included: (a) mean values for the whole responses; (b) standard deviation for the total dataset; (c) individual response for the former round; and (d) IQR for the different criteria. The form was also augmented with additional space for the experts to devise modifications to the indicators or suggest new feedback.

Tables 2, 3 summarize the criteria selected with a breakdown of the results after the second and the third round of consultation.

**TABLE 2:** Criteria consensuses by categories (second round).

Criteria	Category 1: Current situation of the distribution channel	Mean	Mode	Median	Standard deviation	Q1	Q3	Interquartile range (IQR)	Variation coefficient (CV)
1	Structure of the current distribution channel (model 1)	3.64	4	4	0.5045	3	4	1	0.14
2	Structure of the current distribution channel (model 2)	1.18	1	1	0.4045	1	1	0	0.34
3	Structure of the current distribution channel (model 3)	1.27	1	1	0.4671	1	2	1	0.37
4	Current purchase process is being affected by the omnichannel behavior of consumers	3.82	4	4	0.4045	4	4	0	0.11
5	Distribution channel has kept its traditional structure and size	3.64	4	4	0.5045	3	4	1	0.14

	Category 2: Changes in distribution channels								
6	Distribution channels are starting to get shorter because of the disappearance of some retailers	3.55	4	4	0.5222	3	4	1	0.15
7	New online distributors are emerging in the sector	3.91	4	4	0.3015	4	4	0	0.08

	Category 3: Trends and near future of the distribution channel								
8	In the near future, central purchasing offices will have to adopt the model of online retailers	3.09	3	3	0.3015	3	3	0	0.10
9	In the near future, larger firms will take over the supply of the channel	3.18	3	3	0.6030	3	4	1	0.19
10	In the near future, online selling platforms will open physical stores	3.00	3	3	0.6325	3	3	0	0.21

	Category 4: Consequences and adaptations for supply chain agents								
11	Manufacturers and wholesalers should create online platforms	2.64	3	3	0.5045	2	3	1	0.19
12	Manufacturers and wholesalers should strengthen their relationships with retailers	2.64	3	3	0.6742	2	3	1	0.26
13	Integration of large chains and central purchasing offices will help reduce final prices	2.64	2	2	0.8090	2	3	1	0.31
14	Independent retailers should seek partnerships with central purchasing offices	2.45	2	2	0.5222	2	3	1	0.21
15	Independent retailers should seek partners who are online platforms	2.64	3	3	0.5045	2	3	1	0.19
16	Placing value on the personal services offered by professionals by establishing a price	3.55	4	4	0.5222	3	4	1	0.15
17	Developing customer service programs as a way of providing value and differentiating from brick-and-mortar stores.	3.91	4	4	0.3015	4	4	0	0.08
18	Better timeliness, reducing product delivery times	3.91	4	4	0.3015	4	4	0	0.08
19	Using the customer database to develop more efficient customer loyalty programs	3.18	3	3	0.4045	3	3	0	0.13

**TABLE 3:** Criteria consensuses by categories (third round).

Criteria	Category 1: Current situation of the distribution channel	Mean	Mode	Median	Standard deviation	Q1	Q3	Interquartile range (IQR)	Variation coefficient (CV)
1	Structure of the current distribution channel (model 1)	3.82	4	4	0.4045	4	4	0	0.11
2	Structure of the current distribution channel (model 2)	1.09	1	1	0.3015	1	1	0	0.28
3	Structure of the current distribution channel (model 3)	1.18	1	1	0.4045	1	1	0	0.34
4	Current purchase process is being affected by the omnichannel behavior of consumers	3.82	4	4	0.4045	4	4	0	0.11
5	Distribution channel has kept its traditional structure and size	3.73	4	4	0.4671	3	4	1	0.13

	Category 2: Changes in distribution channels								
6	Distribution channels are starting to get shorter because of the disappearance of some retailers	3.73	4	4	0.4671	3	4	1	0.13
7	New online distributors are emerging in the sector	3.91	4	4	0.3015	4	4	0	0.08

	Category 3: Trends and near future of the distribution channel								
8	In the near future, central purchasing offices will have to adopt the model of online retailers	3.55	4	4	0.5222	3	4	1	0.15
9	In the near future, larger firms will take over the supply of the channel	3.27	3	3	0.6467	3	4	1	0.20
10	In the near future, online selling platforms will open physical stores	3.18	3	3	0.7508	3	4	1	0.24

	Category 4: Consequences and adaptations for supply chain agents								
11	Manufacturers and wholesalers should create online platforms	2.73	3	3	0.4671	2	3	1	0.17
12	Manufacturers and wholesalers should strengthen their relationships with retailers	2.91	2	3	0.8312	2	4	2	0.29
13	Integration of large chains and central purchasing offices will help reduce final prices	2.82	2	2	0.9816	2	4	2	0.35
14	Independent retailers should seek partnerships with central purchasing offices	2.55	3	3	0.5222	2	3	1	0.21
15	Independent retailers should seek partners who are online platforms	2.64	3	3	0.5045	2	3	1	0.19
16	Placing value on the personal services offered by professionals by establishing a price	3.73	4	4	0.4671	3	4	1	0.13
17	Developing customer service programs as a way of providing value and differentiating from brick-and-mortar stores	3.91	4	4	0.3015	4	4	0	0.08
18	Bette r timeliness, reducing product delivery times	3.91	4	4	0.3015	4	4	0	0.08
19	Using the customer database to develop more efficient customer loyalty programs	3.45	3	3	0.5222	3	4	1	0.15

**FIGURE 4:**
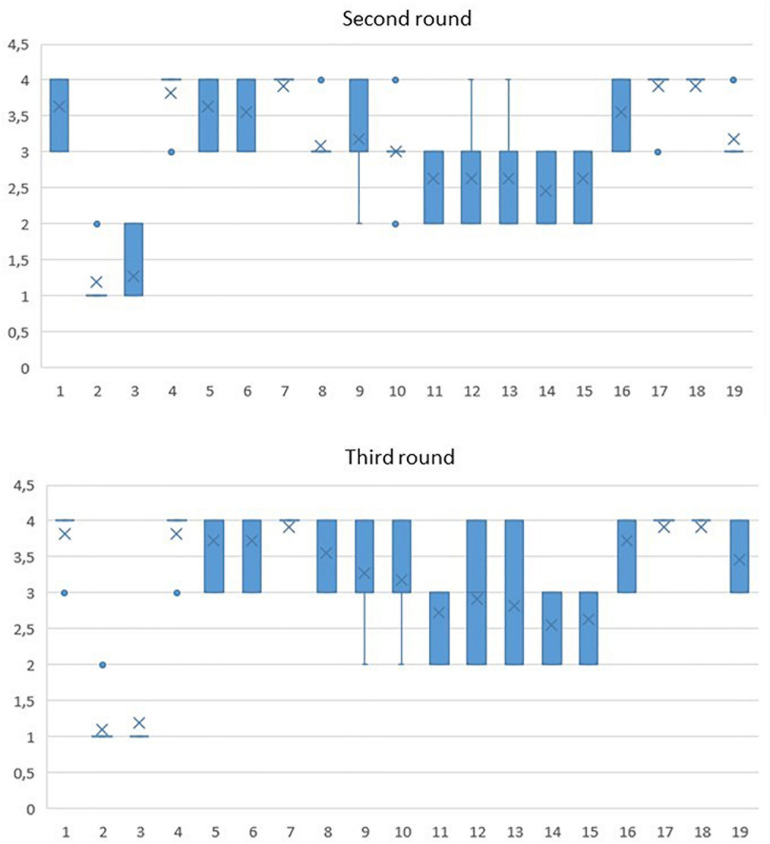
Criteria boxplots after the second and third rounds of consultation.

Figure 4 shows the boxplots for the 19 criteria included. The boxplot is represented by a rectangle where the upper and lower sides correspond, respectively, to the third and first quartiles, while the line contained in the rectangle is the median value. The dashed lines end with whiskers representing the maximum and minimum value of the dataset.

A first glance at Figure 4 reveals that between the second and third round there is an increase in the consensus and a general higher score assignation to the different criteria. The dispersion of the dataset visible from both Figure 4 and Tables 2, 3 shows an improvement from second to third round since the height of the rectangles, including the extensions, decreases.

Criteria 7, 17, and 18 appear to be the most successful criteria registering an outstanding result with 3.91 and the lowest level of dispersion, as illustrated in Figure 4 and Tables 2, 3. The lowest scores have been registered for the criteria 14 and 15 which were assigned 2.55 and 2.64 in the third round with the experts agreeing on their low relevance for this research context.

Table 4 summarizes the four categories selected with a breakdown of the results after the second and third round of consultation. All criteria registered a significant surge both in terms of successful indicators and consensus at the end of the survey, so all criteria deemed satisfactory. Criteria 12 and 13 are deemed satisfactory even in a higher disagreement in the third round (IQR = 2) according to Figure 4. This change is reflected in a higher score even if the standard deviation shows a considerably spread range of opinions around this indicator. Although some experts pointed out that these criteria are important, there is disagreement about their utility.

**TABLE 4:** Summary of results.

Category	Total criteria	Successful criteria after second	Successful criteria after third round
Category 1: Current situation of the distribution channel	5	100%	100%
Category 2: Changes in distribution channels	2	100%	100%
Category 3: Trends and near future of the distribution channel	3	100%	100%
Category 4: Consequences and adaptations for supply chain agents	9	100%	100%
Overall	19	100%	100%
			

The related research based on statistic analysis and the experts’ feedbacks is presented in following subsections.

### Category 1: Current Situation of the Distribution Channel

The key results in relation to this first category are grouped according to the following two main criteria: (1) distribution channel has kept its traditional structure and size, and (2) current purchase process is being affected by the omnichannel behavior of consumers.

#### (Criteria 1–3 and 5) Distribution Channel Has Kept Its Traditional Structure and Size

**FIGURE 5:**
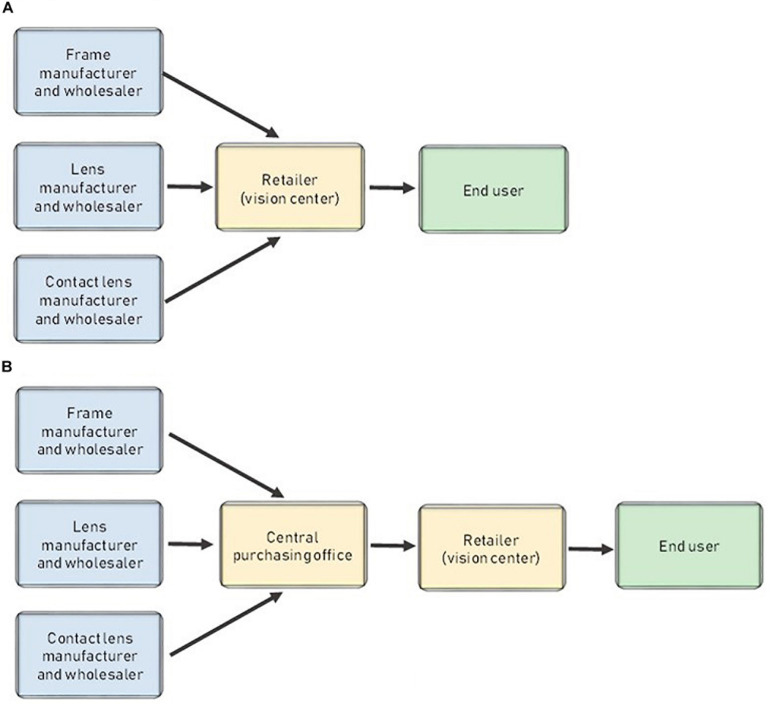
Traditional distribution model in Spain **(A)** and traditional distribution model when there is a central purchasing office **(B)**.

Figure 5 shows the traditional structure of the ophthalmology sector and the whole supply chain in most cases. Channels are short because the manufacturers also act as wholesalers that sell their products (lenses and frames) directly to retailers (vision centers). Consequently, most of their campaigns are business-to-business, and customers do not know about them. In general, the market is highly concentrated, with a few multinationals dominating the entire market.

Retailers are essentially vision centers, at which the presence of at least one optician or eye care professional is mandatory. These vision centers act as retailers while also offering their professional services (eyesight examinations and checks, prevention, treatment of diseases such as glaucoma and cataracts, etc.). Unlike the wholesale market, the retail market is highly fragmented. There are specialized centers (e.g., visual therapy clinics), vision centers that are also hearing centers, and other setups. Fortunately for the existing vision centers, the barriers to entry are high, so traditionally, new entrants are not a genuine threat. The main barriers to entry are initial investment and legal constraints.

Vision centers can be categorized into four groups:

##### Big chains

These companies own centers all over a region, so economies of scale allow them to obtain the lowest prices from manufacturers. One owner controls all centers. Normally, big chains launch communication campaigns and have a single commercial policy for all their stores.

##### Buying groups with a common image/brand

These are groups of independent vision centers that group together for greater bargaining power with manufacturers, typically on prices. They also have a common image/brand as well as some common commercial policies. They have common marketing tools to launch campaigns and attract end customers. In this case, each vision center has a different owner.

##### Buying groups without a common image/brand

These centers also group together for better prices from manufacturers, but their collaboration ends there. They do not have a common image/brand, and they do not develop any common branding strategies. However, owners in this case have more freedom regarding business policies because they control the image of their centers.

##### Independent vision centers

These centers have a single store, and their bargaining power is the weakest. However, they have some advantages such as greater control over the business.

Figure 5 shows the supply chain when buying groups and chains use a central purchasing office. However, the central purchasing office is not an independent agent; it belongs to the retailer.

#### (Criterion 4) Current Purchase Process Is Being Affected by the Omnichannel Behavior of Consumers

Patients who enter a vision center to buy eyeglasses must be examined and must complete a medical history check (family background, allergies, medicines, etc.). After checking the patient’s eyes, the eye care professional must determine the patient’s visual needs and the number of diopters needed for the patient to see perfectly. Once the eye condition has been identified and the way the patient will use the glasses has been determined, the frame and type of lens must be chosen. The lens can be enhanced with additional features and coatings such as tints, antireflective or antidust coatings, and the like. Then, the vision expert orders the lenses from the laboratory, which is also the manufacturer and wholesaler. For the frame, vision centers normally have their own stock. When the optician receives the lenses, they are beveled (cut to fit the frame), assembled into the frame, and given to the patient. Normally, a final check takes place to confirm that everything is correct. This purchase process might change in the future, as discussed. Omnipresence is crucial today for any kind of business, and the ophthalmology sector is no exception. Many consumers prefer to shop online no matter what, attracted by the lower prices that the Internet offers; others would rather visit a physical store.

### Category 2: Changes in Distribution Channels

The main challenge for companies in the sector is to adapt quickly to consumers’ changing buying behavior. Although there are many changes, the key results in relation to the second category are grouped according to the following two criteria: (1) distribution channels are starting to get shorter because of the disappearance of some retailers, and (2) new online distributors are emerging in the sector.

#### (Criterion 6) Distribution Channels Are Starting to Get Shorter Because of the Disappearance of Some Retailers

**FIGURE 6:**
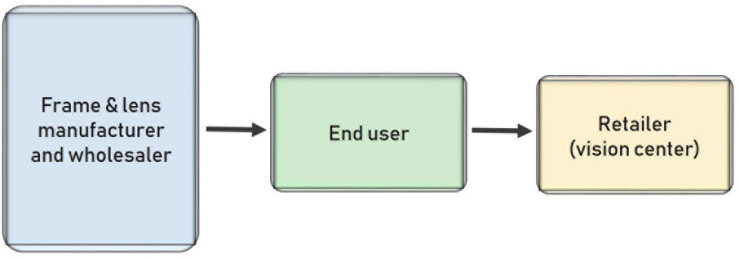
Distribution model when wholesalers sell directly to customers.

Like in other markets, online selling has given wholesalers access to many users that they could not reach in the past. Therefore, wholesalers are tending toward selling their products directly to end users (see Figure 6). The primary reason is cost. If wholesalers can cut out the retailer to reach end customers, they can deduct the vision center’s margin from the final price. This decrease in the final price attracts price-sensitive customers who want to buy these products directly from the wholesalers at a lower price.

The initial investment by the wholesaler is high and consists of the cost of creating a sales platform, forming a new team in the company, and launching business-to-customer communications. Eventually, however, this investment pays off.

This sector in Spain faces a unique challenge. Unlike in other countries, retail stores in Spain must have at least one eye care professional (an ophthalmologist or optometrist). Users cannot order eyeglasses or contact lenses without knowing what their condition is and what prescription they need. The trend is to decouple the service from the product and separate the vision center from the eyeglass provider.

Users seduced by the lower prices offered by wholesalers must perform two tasks. First, they must visit a vision center and pay to be diagnosed. Second, they must visit the wholesaler’s website to pick a frame and the recommended lenses so that their glasses can be shipped to their home. This process takes customers longer, so the lower price is relative because it requires greater effort. The wholesaler can offer eyeglasses directly to end users without the need for any intermediary because it manufactures both the frames and the lenses.

#### (Criterion 7) New Online Distributors Are Emerging in the Sector

**FIGURE 7:**
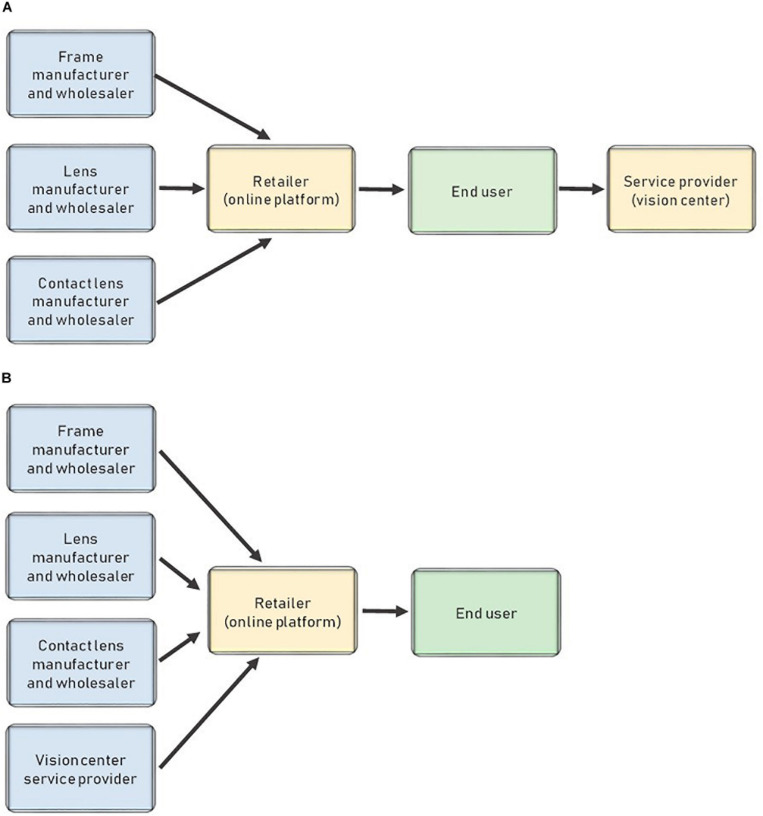
Distribution model for online stores **(A)** and Distribution model of online stores with their own network of vision centers **(B)**.

The previous scenario coupled with the role of the ophthalmologist as a service provider rather than an eyeglass retailer gives rise to a new type of company: online retailers that sell glasses to the public. These online retailers only need a prescription and some lens and frame providers. They then assemble the glasses and ship them to consumers. Figure 7 shows the structure of these new channels.

This new business model is basic and enables two options. First, users can upload photos of themselves and try frames virtually using these photos. Second, using a *home try-on* model, customers can choose different frames from the website that they receive at home. They then choose one and return the others. Finally, after uploading the prescription, customers receive their eyeglasses with their required corrective lenses.

This model represents not only a new way of doing business but also a reduction of the high barriers to entry that previously kept new entrants out of the market. This shift creates an opening for many new companies that now pose a major threat to traditional vision centers, making this market less profitable because of the higher number of competitors.

The main barrier when starting a business is the initial investment in the store. However, by eliminating the costs of opening a physical store, new entrants can enter the market and offer cheap eyeglasses. In this new business model, frames and lenses are sold online by newly appearing companies, and end users must get their eye examination results from a vision center that does not participate in the sale of the glasses or contact lenses.

In Spain, this change would represent an advancement. The online retailers selling these eyeglasses would partner with vision centers. They would then have their own network of professionals to examine customers’ eyesight and issue prescriptions so that customers could purchase their glasses online. In this case, the service and the product would be in the same channel, as illustrated in Figure 7.

In this new supply chain model, eyeglasses are cheaper than in the traditional model for several reasons. First, the agreement between the online platform and the vision centers means that the user does not pay for the eyesight examination or prescription. Second, there is less transparency regarding the origins of the materials, so in some cases, the products might be of a lower quality.

### Category 3: Trend and Near Future of the Distribution Channel

Although regulations are normally more restrictive in the European Union than in the United States market, many trends that have occurred in the United States can also spread to European countries, especially Spain, where there are still no regulations. The key results in relation to the third category are grouped according to the following tree criteria: in the near future, (1) central purchasing offices will have to adopt the model of online retailers, (2) larger firms will take over the supply of the channel, and (3) online selling platforms will open physical stores.

#### (Criterion 8) in the Near Future, Central Purchasing Offices Will Have to Adopt the Model of Online Retailers

**FIGURE 8:**
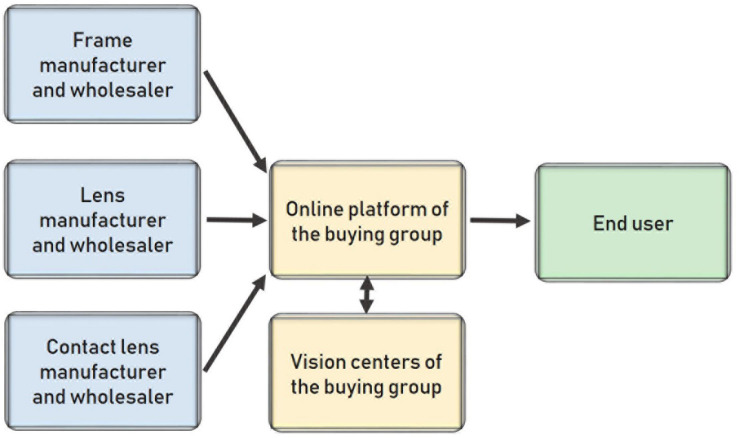
Distribution model when a buying group opens an online selling platform.

In this model, a buying group creates an online platform where it displays frames and rough prices, like online stores. However, the buying group uses its own network of vision centers, so it does not need any partners outside the group itself. This new distribution model resembles the model in Figure 7. The only change is that the vision centers belong to the same group as the online platform. This model is represented in Figure 8.

#### (Criterion 9) in the Near Future, Larger Firms Will Take Over the Supply of the Channel

**FIGURE 9:**
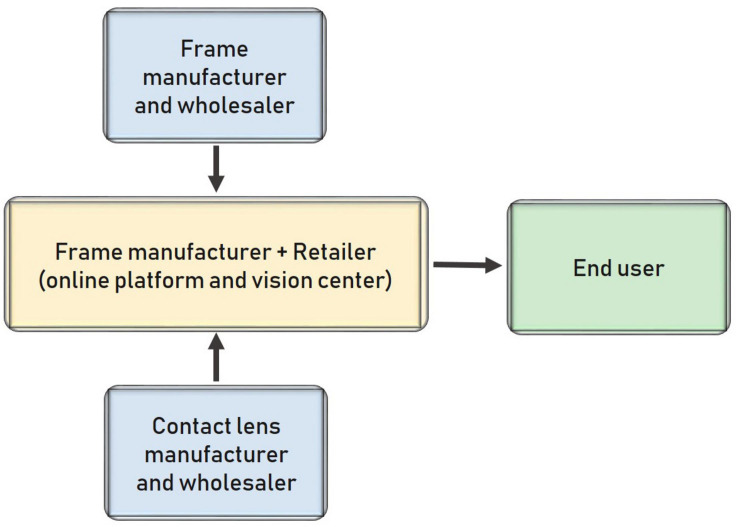
Distribution model where frame manufacturers and retailers are joined.

The next step in the strategy of big companies is insourcing as much as possible if they are solvent enough. Retailers can open their own frame workshops (lenses are harder to manufacture), while manufacturers can open their own vision centers. This new distribution model is depicted in Figure 9. These big companies with insourcing can sell products with total control over the process and without intermediaries adding margins to the final price.

#### (Criterion 10) in the Near Future, Online Selling Platforms Will Open Physical Stores

Most eyeglass and contact lens users are seniors. Traditional vision centers are most effective at capturing these senior users. In addition, eyeglasses are directly linked to health care, and many customers still prefer to visit the shop to touch the product and interact with staff. Therefore, online stores are expected to take a step backward by opening brick-and-mortar stores to reach these customers.

Online stores use the Internet to overcome the financial barriers of opening a physical store to start and grow. However, eventually they must open physical stores to reach population segments that do not use online stores. These companies would insource the service and would have their own network of vision centers with eye care professionals hired inside the company. In this case, these eye care professionals would be employees rather than having their own businesses, so the whole process would stay inside the same company.

**FIGURE 10:**
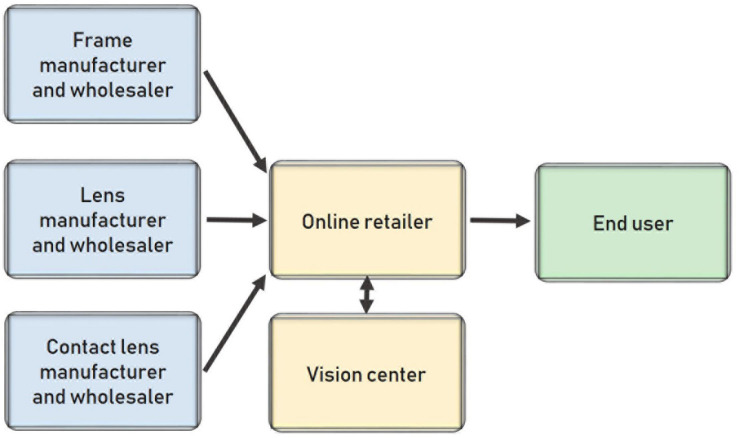
Distribution model when the online platform opens its own vision centers.

Europe normally has more rigid and restrictive laws than the United States. Consequently, online stores are likely to face many difficulties in the future. These online retailers are highly restricted, so opening physical stores can prove effective at avoiding the legal barriers that the current online businesses will face in the future in terms of quality, standards, and regulations. In this case, the new distribution model does not arise as a way of reducing margins and the final price but rather as a way for online retailers to reach segments of the population who do not like shopping online (see Figure 10).

### Category 4: Consequences and Adaptations for Supply Chain Agents

The agents in the supply chain must adapt and evolve to avoid being left behind. The course of future events is still unclear because it will depend on what legislation is passed in the future. In the meantime, there are many ways for agents to compete and adapt in this new era, in which changing consumer habits have led to a new scenario of online stores and short distribution channels. Some of the adaptations are highly positive and can be used as a competitive advantage. The main consequences and adaptations in relation to the fourth category are grouped according to the following four criteria: (1) manufactures and wholesalers, (2) large chains and central purchasing offices, (3) independent retailers, and (4) competitive advantages for traditional retailers.

#### (Criteria 11–12) Manufacturers and Wholesalers

Manufacturers and wholesalers could adopt one of the following two positions. The first would be to sell products at a lower price to end consumers by creating online platforms. Companies adopting this position would lose retailers to distribute their products but would gain end users. The second position would be to eschew this new way of working, reinforcing relationships with retailers, which are considered essential. Companies adopting this position would not gain income from end users but would gain more retailers to distribute their products.

#### (Criterion 13) Large Chains and Central Purchasing Offices

Through economies of scale and an intensive distribution strategy, big chains and buying groups would secure highly competitive prices from providers and could offer relatively cheap products.

#### (Criteria 14–15) Independent Retailers

Physical vision centers would not disappear completely because they perform many activities besides selling eyeglasses. If vision centers stopped selling eyeglasses, only certain types of vision centers would survive. Even if vision centers did not disappear, online retail could be detrimental to ophthalmologists or optometrists by making them expendable. Retailers would have to shift to e-commerce using one of two options. The first would be to join a buying group. By doing so, they would get better prices from suppliers, which would mean better prices for customers. Furthermore, buying groups would have the tools to open online retail platforms. The second option would be to form a partnership with online platforms to attract new customers without needing to join a buying group.

#### (Criteria 16–19) Competitive Advantages for Traditional Retailers

The main agreed competitive advantages are:

(a) Developing customer service programs as a way of providing value and differentiating from brick-and-mortar stores. Developing such an eyewear service plan has tremendous short-term and long-term value. These plans help patients understand the value and worth of their opticians. Patients realize that certain things cannot be done online, such as making adjustments to glasses, and that the services of an expert are necessary.
(b) Better timeliness, reducing product delivery times. It helps with timeliness because vision centers typically take less time than online retailers to prepare glasses.
(c) Placing value on the personal services offered by professionals by establishing a price. Professionals should be advised to charge for their personal services as a symbol of professionalism and premium service.
(d) Using the customer database to develop more efficient customer loyalty programs. Vision centers should focus on building customer loyalty through a loyalty program.

## Termination Criteria of the Delphi Process

**FIGURE 11:**
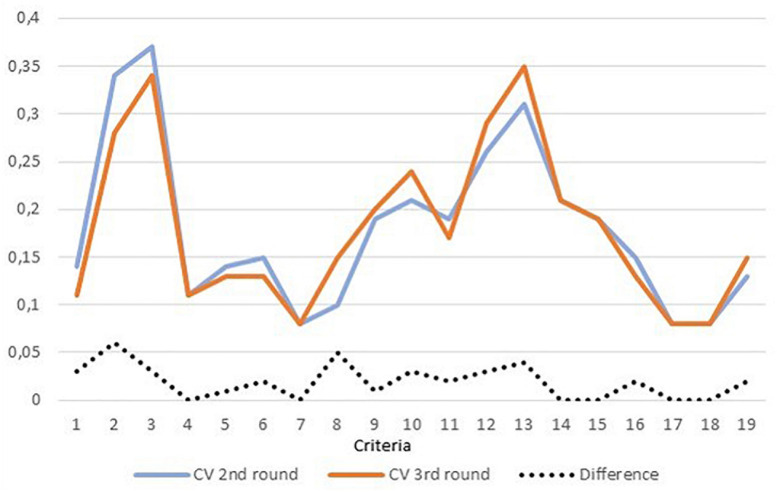
Variation coefficients for the criteria over the second and third rounds of consultation.

As anticipated in section “Consensus,” Dajani et al. (1979) classify as a termination option the achievement of stability in combination with the consensus target fulfillment. Stability is defined as the statistical consistency between two values for the same variables across two rounds of the consultation. In order to be quantitatively assessed, the methodology devised by English and Kernan (1976) is adopted. In section “Consensus,” the methodology devised by English and Kernan (1976) has been introduced in reference to the work by Dajani et al. (1979). Based on the values of mean and standard deviation, the variation coefficient has been calculated and its trend is presented in Figure 11. The dotted line represents the absolute difference between the variation coefficients between the two rounds. English and Kernan (1976) establish that a variation coefficient between 0 and 0.5 is acceptable to consider consensus achieved and hence terminate the process. Firstly, it has to be observed that overall the criteria fulfills the requirement of stability and hence justify the termination after the third round.

## Discussion

Table 4 summarizes the four categories selected with a breakdown of the results based on statistical analysis shown in Figure 4 and Tables 2, 3.

Overall, the majority of the criteria scored over 3 points, while a minority was rated between 2.5 and 2.91 points and were deemed not relevant. On the whole, a significant increase in the consensus rate has been registered moving from one round to the other, raising the number of satisfactory indicators for each round, with an overall success and stability of about 100% after the third round. These 19 criteria identified and accepted by consensus support the idea that the arrival of omnichannel distribution is inevitable in the eye care sector (General Council of College of Optics-Optometrics (GCCOO), 2019). Consumers nowadays tend to combine these digital services with brick-and-mortar establishments to search and shop (Nash et al., 2013; Kang et al., 2019). However, the fact is that traditional channel and physical stores remain the first choice today because new consumers in the digital era still value certain attributes that belong exclusively to physical stores (General Council of College of Optics-Optometrics (GCCOO), 2019).

The increasing trend of shopping online in ophthalmology sector could be one of the reasons for this change in preferences, as anticipated in the work by Khanna and Bhatia (2018). Moreover, the adoption of an omnichannel strategy in ophthalmology sector results in an increase in the number and complexity of customer–firm interactions (Melero et al., 2016) and the use of this integrated system is still hard for both upstream suppliers and downstream retailers as noted Ailawadi and Farris (2017). The criteria which have been judged as less important in this context belong to the category 4 related to the main consequences and adaptations for supply chain agents identified by the experts. Experts argue about the relevance of these criteria throughout all the considered phases. However, many others adaptations have registered the highest scores (developing customer services programs and reducing product delivery times) related to new omnichannel habits of purchasing (Nash et al., 2013; Melero et al., 2016; Kang et al., 2019).

## Conclusion

### Theoretical Implications

The Internet has transformed the buying behavior and habits of customers and the arrival of omnichannel distribution is inevitable in the eye care sector (Kang et al., 2019). In this sector, the single distribution model has been divided into a range of more complex and interrelated distribution models. It is no longer possible to talk of a rigid distribution supply chain for eyeglasses or contact lenses.

The results confirm that traditional intermediaries face difficulties to avoid being squeezed out of the sector because of shortening distribution channels and the entry of new online intermediaries with service integration models as noted Ailawadi and Farris (2017).

On one hand, this work identify future scenarios and define actions that different retailers can take to strengthening the competitiveness of offline retail through specializing its own uniqueness (Sopadjieva et al., 2017; Fisher et al., 2018). Retailers should take the change indecision into account when planning their marketing mix strategy process when consumers gain more online buying experience (Melis et al., 2015). Actions such as the development of digitized and integrated customer service programs or a reduction in product delivery times strengthen the customer’s relationship with the traditional retailer and improve its positioning with respect to the online channel in the early stages of the buying process related to need of recognition and information search (Puccinelli et al., 2009). However, and what is more relevant, the intervention of the healthcare professional in the last stages of the buying process related to evaluation, purchase and post-purchase (Puccinelli et al., 2009), adds value to the omnichannel and generate a customer dependence on the offline store that has to be taken in advantage by traditional retailers to enhance the offline buying experience of omnichannel customers. An important motivation to keep an offline channel is indeed that this personal service can increase customer satisfaction and loyalty and help to retain existing customers.

On the other hand, the study shows that manufacturers and wholesalers concentrate and integrate online and offline channels much better in such a way that they may feel less urgency than retailers to adapt, according to the idea that offline and online channels can be consistent, concurrent and compatible (Gabisch and Gwebu, 2011; Melero et al., 2016).

### Managerial Implications

Most of the changes in the distribution channels are caused by the following factors: (1) the disappearance of retailers, eliminating unnecessary margins and enabling lower final prices for end consumers; (2) the appearance of new agents attracted by the profitability of the sector and enabled by online retail’s removal of barriers to entry; and (3) buying groups’ adoption of the online platform model to sell products and compete with online retailers.

If Spain evolves the same way as other economies have, the next steps will be as follows: (1) online channels will open physical stores to attract customers who cannot be reached through the Internet; (2) big companies will insource as much of the channel as possible. Likewise, if the shift toward omnichannel continues, integrated communication must be parallel to distribution. Information sources and up-to-date websites are exceedingly relevant in this sense, but the coordination between wholesalers and retailers in sharing product information is perhaps even more important. New technology, such as online platforms, kiosks, and applications to try on eyeglasses anywhere, will soon be implemented to revolutionize the sector and introduce a new challenge.

### Limitations

In this subsection, we enlist a set of limitations of this research, which provides opportunities for future work. This research has been conducted to have a better understanding of how manufacturers and intermediaries have to face on new habits of purchasing. The novelty of this paper is that it addresses an academic research gap by studying the distribution channels for regulated products and services such as health products in ophthalmology sector that required a closer professional service. Additionally, the proposed methodology (Delphi method) allowed to gather experts’ feedback from different professional backgrounds (manufacturers, wholesalers and retailers), hence approaching the research from different standpoints. This study has an exploratory character, so the statements, recommendations and conclusions made in this work should be taken into account with cautions. Although the size of the applied sample has been adequately justified according to the theoretical framework, in future works it would be interesting to repeat the analysis process with a larger sample to test the internal validity and reliability of the results. Likewise, it would also be appropriate to enrich the sample and include experts with an academic profile to identify new criteria or relevant factors to better understand the evolution of the supply chain in the ophthalmology sector.

Finally note that this study has focused solely on an analysis of supply. A future line of research focused on the analysis of demand and consumer experience with respect to the role of the health professional will complement the results of this work.

## Data Availability Statement

The datasets generated for this study are available on request to the corresponding author.

## Ethics Statement

Ethical review and approval was not required for the study on human participants in accordance with the local legislation and institutional requirements. Written informed consent from the participants was not required to participate in this study in accordance with the national legislation and the institutional requirements.

## Author Contributions

LR and RS contributed to the conceptualization, methodology, formal analysis, investigation, resources, writing the original draft, and visualization. LR contributed to the supervision and project administration.

## Conflict of Interest

The authors declare that the research was conducted in the absence of any commercial or financial relationships that could be construed as a potential conflict of interest.
